# Effect of Breast Cancer Treatment on Dietary Vitamin Intake Levels

**DOI:** 10.3390/ijerph18010019

**Published:** 2020-12-22

**Authors:** María Morales-Suárez-Varela, Amparo Ruiz Simon, Salvador Blanch Tormo, Ismael Pastor Climente, Maximino Redondo Bautista, Isabel Peraita-Costa, Agustin Llopis-Morales, Agustin Llopis-Gonzalez

**Affiliations:** 1 Unit of Public Health, Hygiene and Environmental Health, Department of Preventive Medicine and Public Health, Food Science, Toxicology and Legal Medicine, School of Pharmacy, University of Valencia, 46100 Burjassot, Spain; ipecos@alumni.uv.es (I.P.-C.); allomo3@alumni.uv.es (A.L.-M.); agustin.llopis@uv.es (A.L.-G.); 2 CIBER of Epidemiology and Public Health (CIBERESP), Institute of Health Carlos III, 28029 Madrid, Spain; 3 Medical Oncology Department, Fundación Instituto Valenciano de Oncología (IVO), 46009 Valencia, Spain; amparoruizsimon@gmail.com (A.R.S.); salvabtormo@gmail.com (S.B.T.); 4 Pharmacology Department, Fundación Instituto Valenciano de Oncología (IVO), 46009 Valencia, Spain; ipastor@fivo.org; 5 Red de Investigación en Servicios de Salud en Enfermedades Crónicas (REDISSEC), Hospital Costa del Sol, 29600 Marbella, Spain; mredondo@hcs.es; 6 Department of Biochemistry, Molecular Biology and Immunology, School of Medicine, University of Málaga, 29071 Málaga, Spain

**Keywords:** breast cancer, micronutrients, epidemiology, diet

## Abstract

Breast cancer is the most common tumor among women, representing the second cause of cancer deaths in women. Treatment with chemotherapy negatively interferes with nutritional status. The intake of vitamins before, during and after treatment in a pilot cohort of women with non-invasive breast cancer (type I, II) treated at the Valencian Institute of Oncology (IVO) is evaluated. A 3-day anthropometric and nutritional assessment was performed using the DIAL program. Nutritional intake is compared with the values of Estimated Average Requirements (EAR) and Dietary Reference Intake (DRI) provided by the United States Department of Agriculture (USDA) and the European Food Safety Authority (EFSA). There is an overall decrease in vitamin intake during treatment which worsens at the end of said treatment. The decrease is significant in the case of vitamins B_2_ (*p* = 0.006), B_3_ (*p* = 0.042), B_5_ (*p* = 0.001), and B_8_ (*p* = 0.021). The relative risk during and after treatment increases with respect to the reference timeframe, before treatment. Deficit risks are statistically significant in the case of vitamins B_5_ (*p* = 0.001), B_8_ (*p* = 0.001) and B_12_ (*p* = 0.001). Decreased vitamin intake during treatment suggests a negative change in the patients’ dietary behaviors during this time. Nutritional intervention and support may be beneficial to optimize overall dietary intake and maintain compliance with EAR and DRI for patients during a time in which adequate nutrition is important.

## 1. Introduction

Breast cancer is the most common tumor among women worldwide, accounting for 16% of all female cancers. It is estimated that 519,000 women died of breast cancer in 2004 and, although this cancer is considered a developed world disease, the majority (69%) of deaths occur in developing countries [1].

Nutrition plays an important role in cancer patients, finding a high prevalence of malnutrition among these patients [2]. A state of malnutrition supposes an increase in the risk of mortality and morbidity, lengthening the recovery process of the patient and therefore the sanitary cost [3,4].

The drugs used in this type of treatment are called chemotherapy or antineoplastic, designed to destroy cells while they divide. The faster they divide, the more sensitive they are to treatment. Over time, this results in a decrease in the size or disappearance of the tumor [5].

For many patients, cancer treatment makes it difficult to obtain adequate nutrition [6]. Changes in the nutrition of women during chemotherapy, that contribute to the development of malnutrition, have been observed. These modifications have been evidenced more quantitatively than qualitatively. Cancer treatment often causes dysgeusia, an alteration of one’s sense of smell and taste [7,8] decreasing both the desire to eat and the enjoyment of eating, which can lead to weight change and nutritional deficiencies [9]. Cancer patients also report early satiety and food aversions as a result of treatment [10] as well as changes in energy levels. Cancer treatment also has associated side effects such as nausea, oral mucositis and dry mouth, among others, that can affect dietary intake and nutritional status [11]. 

Given that both the type of cancer diagnosed and oncological treatments affect the nutritional status of patients, nutrition plays a fundamental role in the progression and treatment of the disease. Antineoplastic treatments can produce a micronutrient deficit, hence the importance of nutritional therapy, to reduce the toxicity of treatments and thereby improve tolerance and the quality of life of the cancer patient [12].

Cancer and cancer therapy are associated with oxidative stress and disorders in the balance of the antioxidant system, this could be involved in the toxicity and side effects associated with treatment with antineoplastic agents. Vitamins participate as cofactors in a series of enzymatic reactions and some also have an antioxidant effect [13]. Many pathological processes are caused by a deficit of vitamins. It has been seen, that in breast cancer a correct intake of certain vitamins could have a beneficial, protective effect and positively influence the recovery of patients during and after treatment with chemotherapy [13,14,15,16].

Cell culture studies and animal models establish that vitamin A significantly reduces breast cancer [17]. Laboratory studies with animals claim that consumption of vitamin D affects the growth of cancer cells [12]. Dietary administration of vitamin E has been shown to significantly reduce tumor volume, suppress tumor growth and multiplicity of spontaneous murine mammary cancer [18,19]. There is controversy regarding the beneficial/harmful effects of vitamin B1 consumption [20]. In vitro studies claim that vitamin B3 maintains DNA stability [21]. Some observational studies establish that the consumption of vitamin B_12_ and B_9_ could be associated with a reduction in the incidence of breast cancer [22,23,24,25]. Studies and laboratory tests suggest that the use of antioxidants, including vitamin C, during chemotherapy treatment is related to tumor cell protection and reduces the efficacy of treatment [26,27].

The objective of this study was to assess the evolution of the dietary intake of vitamins during the three phases of chemotherapy treatment in women with breast cancer to establish if there could be a change in the dietary intake pattern, behavior, or habits of the patients during the different stages of treatment that may benefit from a nutritional intervention.

## 2. Materials and Methods 

### 2.1. Sample

The study was carried out in a pilot cohort of women diagnosed with non-metastatic breast cancer (type I, II) that are treated at the Valencian Institute of Oncology (IVO). The study protocol was approved by the IVO Ethics Committee with registry number (2013/23) and follows the rules of the Helsinki Declaration of 1975.

All women who met the inclusion criteria of: (1) being recently diagnosed with non-metastatic breast cancer (Stage I and II); (2) having not yet received any treatment for given cancer and (3) going to receive treatment with chemotherapy at the IVO were offered the opportunity to participate in the study. No exclusion criteria regarding age, preexisting conditions etc. were established, and women were only not included if they did not agree to participate. The agreement to participate was formalized by signing an informed consent. The recruitment period was from the second semester of 2015 to the first semester of 2017.

**Figure 1:**
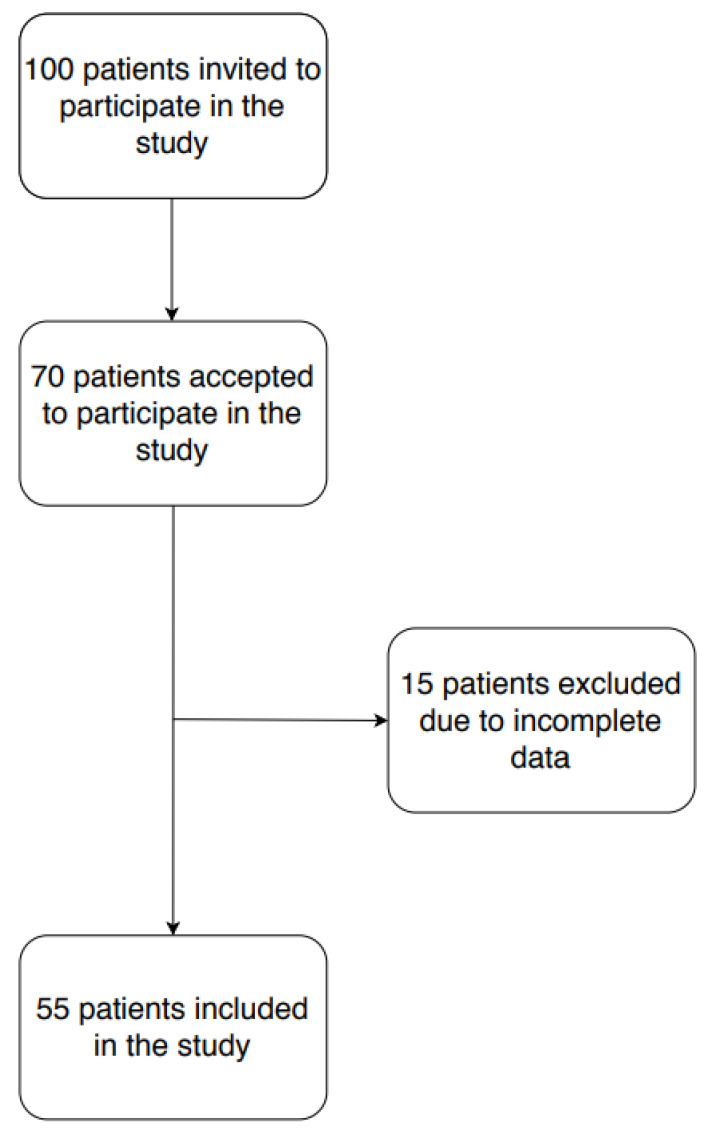
Flow chart selection of women with breast cancer participating in the study.

Of the 100 women who were invited to participate, 70 accepted. The 70 participating women underwent three nutritional assessments: the first after the diagnosis, but before the treatment, the second in the middle of the treatment and the third after the end of the treatment (Figure 1).

For this study, only those women who completed the follow-up in the three assessment periods of the cohort were included. Women who did not return all 3 surveys or returned surveys with incomplete information were excluded. Finally, 55 women were included in the study (Figure 1). Data have also been collected on the age, weight and physical activity of the patients.

### 2.2. Food and Beverage Records

The assessment of food intake was carried out using a 24-h dietary questionnaire for three consecutive days in the three stages of the study: before, during and after the treatment, which in this study involved a total of 111 food surveys. The questionnaires were filled out by the patients themselves after previous instruction, without making dietary recommendations. In order to obtain the nutritional information of the food, the DIAL computer program [28] has been used, which allows identification of the energy and main macro and micronutrients contained in the food consumed.

### 2.3. Evaluation of Misreporting

The nutritional intake of micronutrients, specifically vitamins, was compared with the Reference Daily Intake (RDI), also known as Recommended Dietary Allowance (RDA) established by the EFSA [29] and the EAR established by the USDA [30].

The EAR is the level of daily intake of a nutrient that is estimated to cover the requirements of half of healthy individuals according to age and sex. The RDI is the average intake level of a nutrient that is considered sufficient to meet the nutritional needs of almost all (97–98%) individuals in a population and the current levels of physical activity and lifestyle of the population are taken into account.

For the nutrients where an EAR or RDI value has not been established, Adequate Intake (AI) has been used. The AI is established when there is still not enough scientific evidence to determine the RDI. It is based on estimating the amount of a nutrient consumed by a group of healthy people and assuming that the amount they consume is adequate to promote health. The AI according to age and sex is set at a level to cover or exceed the needs of almost all people in a specific age and sex category [31,32].

### 2.4. Statistical Analysis

A database was made with all the information collected: personal data, clinical data and vitamin intake in each of the periods of the study. The mean and standard deviation of each of the vitamins studied were calculated in the three moments of the study and compared with the analysis of variance (ANOVA) test with Bonferroni correction. The percentage of EAR was calculated in each of the periods studied and compared by the Chi-square test (χ^2^) with the Bonferroni correction. Data are presented as means ± standard deviations (sd) for continuous variables and as frequencies for discrete variables. One-way analysis of variance (ANOVA) was performed to investigate differences between groups in continuous variables while the Chi-square test (χ^2^) was used to explore differences between groups in discrete variables with Bonferroni correction. The Hazard Ratio (HR) and its 95% Confidence Interval (95% CI) were calculated for each of the vitamin intakes, taking as a reference level the pre-treatment intake and the Trends Manteal test was applied. In all comparisons a *p* value below 0.05 was considered statistically significant. All statistical analysis was performed using the Statistical Package for the Social Science (SPSS) IBM SPSS Statistics for Windows, Version 22.0 (IBM Corp., Armonk, NY, USA). 

## 3. Results

Table 1 shows the main characteristics of the cohort. A total of 55 women with breast cancer participated in the all three periods of this cohort study. The women are in an age range between 30 and 79 years old, average age of 51.49 ± 11.17 years. The Body Mass Index (BMI) is between 18.9 and 35.8, the average is 25.52 ± 4.83, which is within the overweight category. The Physical Activity Level (PAL) performed by women is between 1.20 and 1.64, the average woman performs a light physical activity of 1.48 ± 0.19.

**Figure 2:**
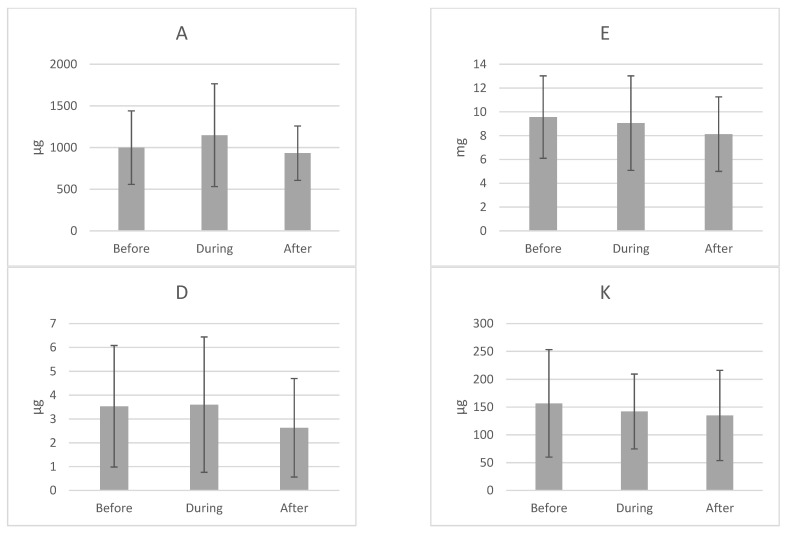

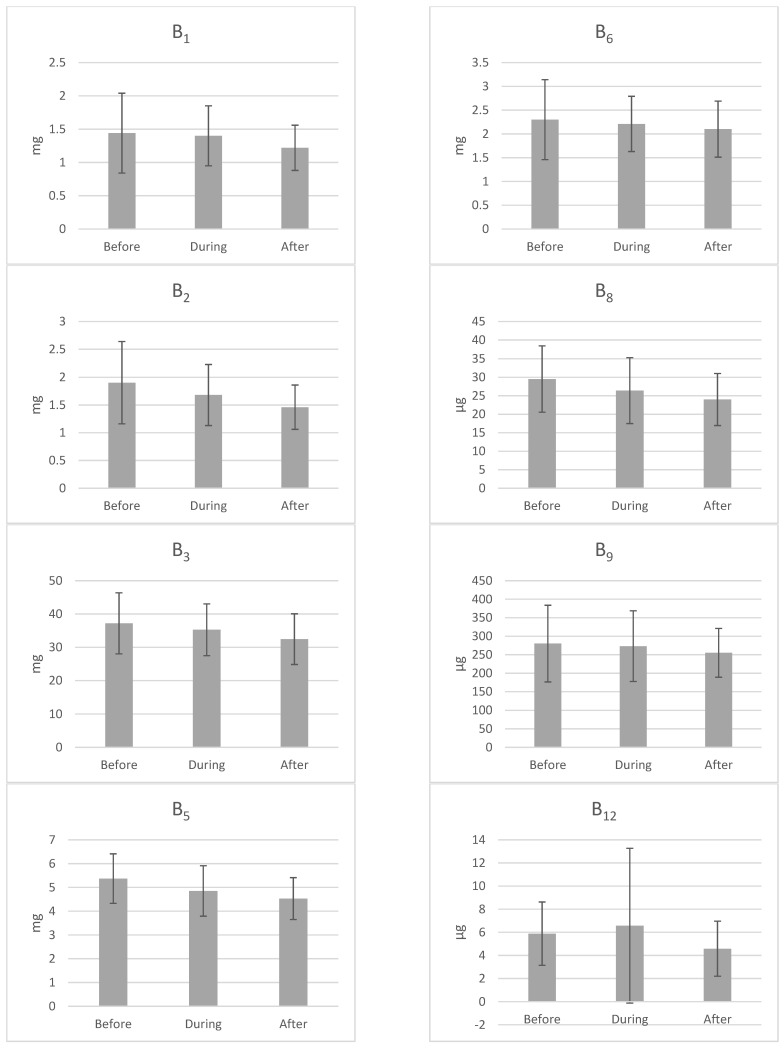

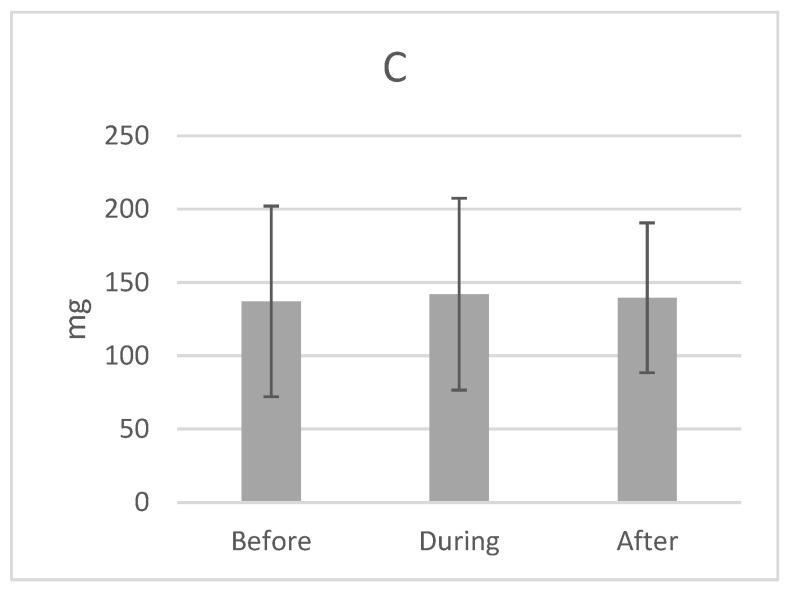
Mean ± standard deviation of vitamin intake in the three stages of the study.

**Figure 3:**
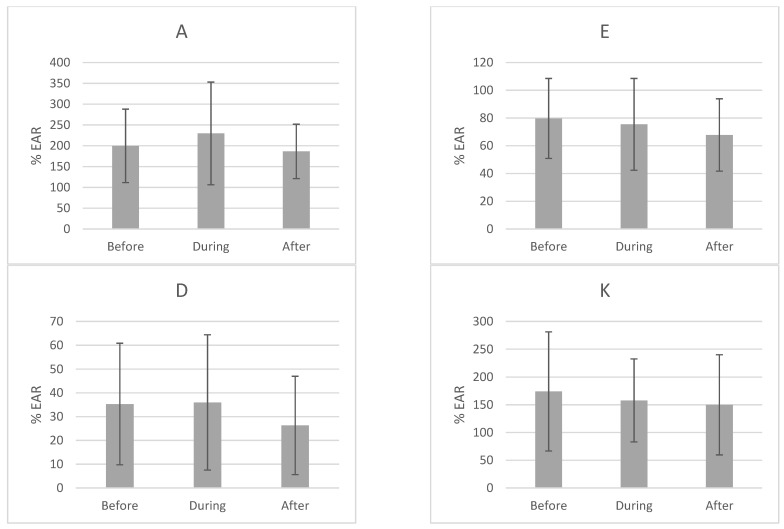

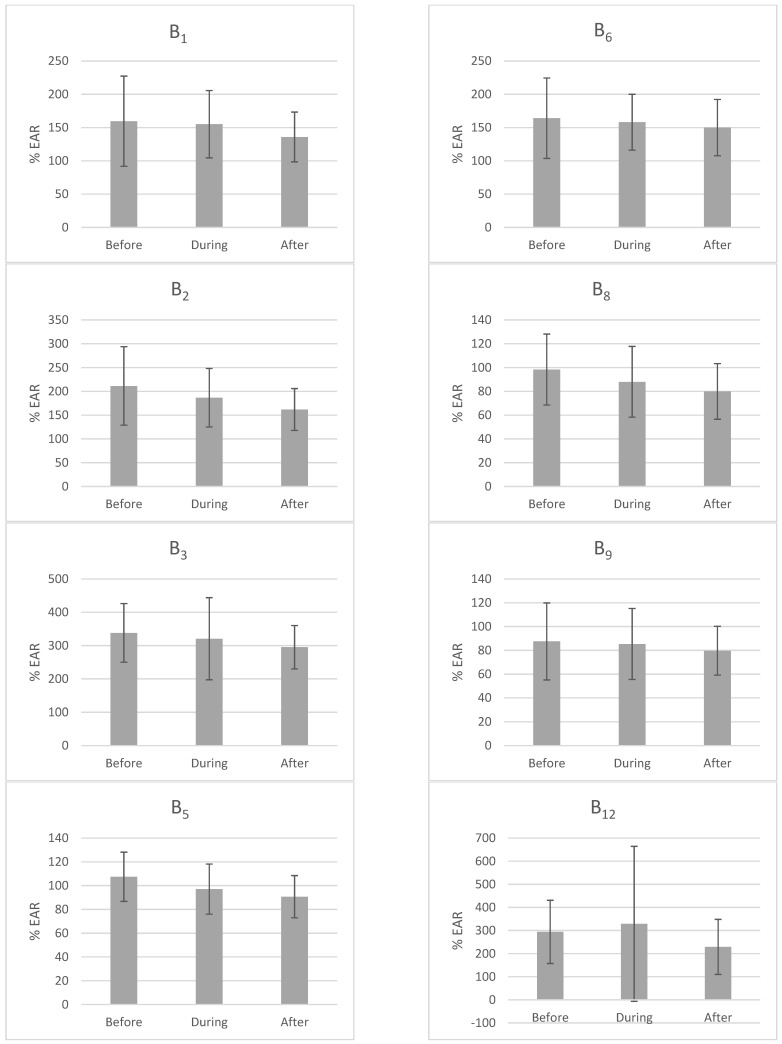

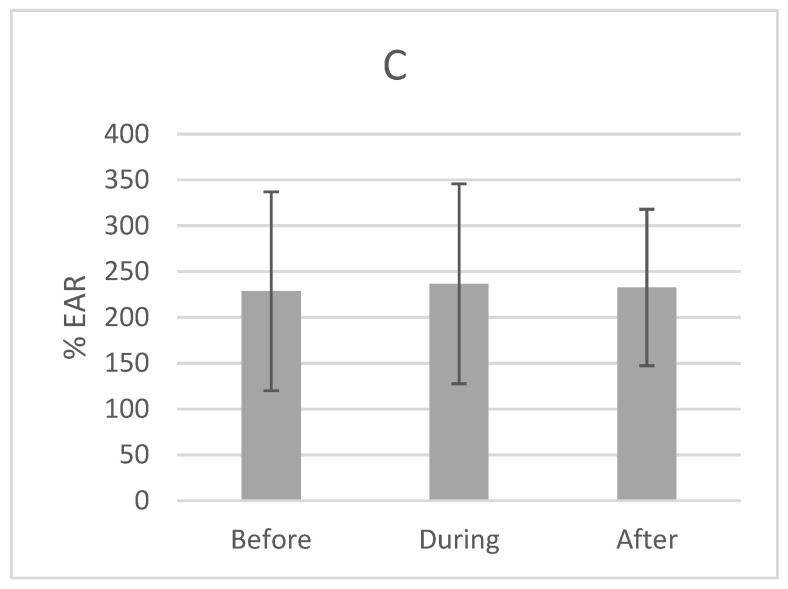
Percentage of estimated average requirement of vitamin intake in the three stages of the study.

Table 2, Figure 2 and Figure 3 show the values of vitamin intake in the three stages of the study; before, during and after treatment. The average intake of most vitamins remains at or above the EAR values: A, K, B_1_, B_2_, B_3_, B_6_, B_12_ and C. In the cases of vitamins A and B_3_, the maximum levels of intake reach beyond the toxic limit at some point during the study. In contrast, for vitamins D, E, B_5_, B_8_ and B_9_ the intake is lower than recommended. In addition, the vitamin intake that at the beginning of the treatment did not comply with the EAR, does not comply with it at the end of the treatment, the intake is actually even more reduced. If we look at minimums and maximums, we observe that there are women who do not comply with even 50% of the EAR.

All vitamins are ingested in less quantity after starting treatment. Despite the results, only statistically significant changes were observed in the intake of vitamins B_2_ (*p* = 0.006), B_3_ (*p* = 0.042), B_5_ (*p* = 0.001) and B_8_ (*p* = 0.021).

Table 3 shows the percentages of intake below the EAR, of each of the vitamins studied in each of the moments of the study. We can observe that the consumption of vitamins is lower than the EAR with greater frequency during and/or after the treatment, with the exception of vitamin C, where its consumption increases during and after the treatment. We found statistically significant decreases, when comparing the three periods, in the intake of vitamin B_5_ (*p* = 0.002), B8 (*p* = 0.001) and B12 (*p* = 0.001).

The risk of an intake lower than the EAR of each vitamin is expressed with the hazard ratio (HR) and its confidence interval at 95% (CI95%) taking as a reference level the intake prior to treatment. A pattern is observed in which the risk increases during and/or after the treatment of vitamin intake deficit. Except in vitamin C, in which we can observe that the relative risk during and after treatment decreases with respect to the reference. Deficit risks are statistically significant in the case of vitamins B_5_ (*p* = 0.001), B_8_ (*p* = 0.001) and B_12_ (*p* = 0.001).

## 4. Discussion

Currently, higher morbidity and mortality rates are observed in cancers such as breast cancer [4]. In Spain, about 26,000 cases of breast cancer are diagnosed each year, which represents 30% of all female cancers in the country. The Spanish Association Against Cancer (AECC) states, like the present study (Table 1), that most cases are diagnosed between 35 and 80 years, with most between 45 and 65 [33]. 

In the study, BMI and PAL were evaluated as influencing factors (Table 1). It is observed that the average woman who is diagnosed with breast cancer is overweight (BMI ≥ 25), as defined by WHO and engages in sedentary or light physical activity (PAL = 1.40–1.69), as established by FAO in 2001 [32]. Different studies claim that women who are overweight or obese have a higher risk of being diagnosed with breast cancer [34,35]. Therefore, the practice of physical activity becomes a preventive strategy for breast cancer [36].

The purpose of the dietary assessment in this study was to identify the prevalence of inadequate vitamin intake and its evolution during and at the end of chemotherapy treatment. The results reflect a diet imbalanced in vitamins from the beginning, which worsens during treatment and may have negative consequences in the development of the disease. However, a high consumption of certain vitamins is also observed (Table 2).

An adequate nutritional intake in general and relating to vitamins in particular, allows for the maintenance of the body composition and cellular functions of the organism. Alterations in nutritional intake suppose a risk of malnutrition and consequently, an increase of the risk of infections, and of recovery of the patient. Both a state of deprivation and a state of hypervitaminosis can lead to health problems and their presence is even more important in the case of pathologies such as cancer, since they have been found to be cofactors in many enzymatic reactions as well as having an antioxidant effect [31].

To date, there are very few studies that evaluate the evolution of the intake of vitamins in patients with breast cancer. Most studies have evaluated the intake of food using questionnaires of frequency of consumption or have evaluated the intake of certain vitamins in particular, such as vitamin B_9_ or vitamin C. Two previous studies conducted in Brazil on women during chemotherapy treatment have obtained results different from ours. One finds a consumption of vitamins A, B_3_ and B_6_ below the recommendations and vitamin C above [37]. The second also finds a significant reduction in intake and a high prevalence of inadequacy for vitamins B_3_, B_2_, B_1_, B_6_ and C [11].

Vitamin A is related to the regulation of the growth and specialization (differentiation) of human body cells [17]. Through the regulation of the expression of more than 500 retinoid-sensitive genes (including several genes involved in the metabolism of vitamin A), the retinoic acid isomers play major roles in cell proliferation and differentiation [38]. Studies in cultured cells and animal models have demonstrated the ability of retinoids to significantly reduce carcinogenesis in the skin, breast, colon, prostate, and other sites [17].

In this study, patients ingest vitamin A above the EAR and surpassing the DRI, some have even reached the UL (Table 2). Exceeding the limit of toxicity can lead to a state of hypervitaminosis, an intoxication that includes symptoms such as: irritability, headache, anorexia, diplopia, alopecia, joint pain, liver disorders, hemorrhages [12]. Despite this, although the maximum levels are close to the UL, this does not necessarily mean that there is a risk of toxicity, since only a three-day diet has been evaluated at each stage of the treatment, and the requirements may be higher due to the disease and the treatment. Nor should we forget that in the Valencian Community a Mediterranean diet pattern is followed with a high consumption of fruits and vegetables highlighting among them oranges that can increase the intake of vitamin A [39].

Different authors reported a protective role of endogenous vitamin D status in cancer disease [4,40,41,42]. This protective effect of vitamin D is explained by two cellular mechanisms [43]. Laboratory and animal studies show that vitamin D may affect cancer cell growth, apoptosis, and tumor angiogenesis at cellular level [44]. Other studies show that vitamin D supplementation is associated with a decrease in pain caused by bone metastasis of breast cancer and an improvement in quality of life. Adequate intake of vitamin D improves bone mineral density [12]. On the contrary, vitamin D deficiency has been reported to be associated with poor/fair health status, significantly associated with comorbidity (obesity, HTA) and lower level of cholesterol. Although the association of vitamin D level with breast cancer was uncertain, free of cancer survival rates seems to decrease [45,46]. Although in our study there is a notable deficit in the intake of vitamin D (Table 2), it must be borne in mind that this vitamin is also synthesized in the skin. Our study has been carried out in the Valencian Community, an area with a Mediterranean climate. Therefore, it is expected that patients have enough sun exposure to cover this deficit.

Tocopherol and tocotrienols belong to the vitamins E family, with well-known antioxidant properties that are important for protecting polyunsaturated fats from peroxidation [47]. A review suggests a possibility of combined therapy for breast cancer in order to improve the therapeutic response and to lower the toxicity associated with high dose of doxorubicin [48]. Toxicity and resistance to standard drug treatments limits the effectiveness of chemotherapy, highlighting an urgent need for development of potent anticancer drug with reduced toxicity as new treatment strategies. Selected tocopherol, such as tocorinol forms as well as metabolites [49,50] and synthetic derivatives, have been reported to have antitumor and anti-inflammatory activities [51,52]. Dietary administration of vitamin E has been shown to significantly reduce tumor volume, suppress tumor growth and multiplicity of spontaneous murine mammary cancer [18,19]. In the present study despite not finding statistically significant differences, it can be observed that the consumption of vitamin E during the three stages studied is lower than the EAR, even its consumption is still lower during and at the end of the treatment (Table 2 and Table 3).

It has been seen that cancer cells that divide rapidly have a high thiamine (B1) requirement [53]. There are studies that indicate that, although thiamin supplementation is common in cancer patients to prevent deficits, a high amount of thiamine could stimulate the growth of some malignancies [20]. Even so, there are still no studies in humans that support this theory. In our study, we can observe that women have a high consumption of vitamin B_1_ both before, during and after treatment, although with statistically insignificant results (Table 2).

Riboflavin (B_2_) is a precursor of coenzymes, flavin adenine dinucleotide (FAD) and mononucleotide flavin (FMN). These enzymes play an important role in the metabolism of other vitamins: B_6_, B_3_ and B_9_ and a lack of this vitamin could affect many enzyme systems. In the present work, the consumption of vitamin B_2_ exceeds the EAR and DRI in the three stages of the study with statistically significant results (Table 2).

Niacin (B_3_) and its nicotinamide derivatives are dietary precursors of adenine dinucleotide nicotinamide (NAD), which can be phosphorylated (NADP) and reduced (NADH and NADPH). Cell culture studies (in vitro) provide evidence that the NAD content influences the mechanism that maintains genomic stability. The loss of genomic stability, characterized by a high rate of damage to DNA and chromosomes, is a representative signal of cancer [21]. The sources of this vitamin are: meat, especially organs, fish, fatty foods, whole grains, legumes, dairy products and eggs. It is recommended to take caution when ingesting high amounts of this vitamin as an intoxication with vitamin B_3_ can lead to an increase in blood sugar levels, liver diseases that develops with a yellowish color of the skin, general itching and development of ulcers. We have seen that in our study (Table 2) there are patients who exceed the toxic limit of intake of this vitamin in the three stages of the study, therefore, caution must be taken to prevent poisoning.

A deficiency of B_5_ is very rare in humans and has only been seen in cases of severe malnutrition. In our study (Table 2), although significant changes were observed in the intake of B_5_, they did not fall far short of the EAR recommendations. We see that vitamin B5 consumption is statistically significantly lower than EAR more frequently during and after treatment. Therefore, a pattern is observed in which the risk increases during and after the treatment of vitamin B5 intake deficit (Table 3).

Other B vitamins, such as B_6_, B_12_ and folate (B_9_), are important coenzymes for DNA integrity and stability, as they participate in nucleotide synthesis and DNA stability. Deficiencies in B vitamins may promote carcinogenesis. High intakes of vitamin B_6_ have been associated with a reduction in the risk of developing breast cancer [54]. Low folate diets can lead to the deterioration of DNA synthesis and its methylation which can result in the activation of protooncogenes and rearrangement and instability of chromosomes, all capable of promoting the development of cancer [55]. In a study carried out in Norway on female survivors of breast cancer, a folate intake was also observed to be below the recommendations [15].

Although no associations have been found regarding the reduction of mortality risk from breast cancer, it has been seen that B_12_ may have a protective role in those women who consume alcohol [56,57].

A poor vitamin B_12_ status has been linked to an increased risk of breast cancer in some, but not all, observational studies. There is a need to assess whether supplemental vitamin B_12_, together with folic acid, could help reduce the incidence of breast cancer [22,23,24,25]. Regarding the results obtained in the present work, despite not finding statistically significant differences between the three stages studied, we can affirm that the consumption of vitamin B_12_ by these women complies with the EAR and DRI established (Table 2). In spite of this, we do find statistically significant results in that the consumption of vitamin B_12_ is reduced during and after chemotherapy treatment compared to that consumed before treatment. Therefore, a pattern is observed in which the risk increases during and after the treatment of vitamin B_12_ intake deficit.

Vitamin C is a water-soluble vitamin with an antioxidant effect. Its main functions are to act as an antioxidant and enzyme cofactor [58]. In the case of women diagnosed with breast cancer, the consumption of vitamin C, both dietary and supplements, has been associated with a reduction in the risk of mortality from breast cancer [14]. Laboratory studies and randomized trials suggest that the use of antioxidants, including vitamin C, during chemotherapy, may have a protective effect on tumor cells and decrease the efficacy of treatment [26,27]. However, in vitro experiments revealed that at a very high dose vitamin C can act as a prooxidant on cancer cells, demonstrating cytotoxicity in tumor cells without similar effects in normal cells [59,60].

In this study, despite not having statistically significant results, it is observed that the consumption of vitamin C is higher than the EAR and DRI established for women, but not higher than UL (Table 2). The consumption of vitamin C increases during and after the treatment with chemotherapy in the studied patients. Therefore, the risk of an intake lower than the EAR during and after treatment decreases with respect to the reference, before treatment (Table 3). The fact that vitamin C consumption is higher than EAR and DRI and that it increases during and after treatment with chemotherapy could be due to the fact that the study has been carried out within the Valencian Community, in a Mediterranean diet area where between 45–50% of Spanish oranges and tangerines are produced [61].

This study presents some important limitations that must be addressed. While the aim of this study was more focused on the patients’ dietary behavior and habits and determining if there was a change in vitamin intake rather than on specific vitamin levels in the body, it cannot be assumed that an adequate dietary vitamin intake would guarantee adequate vitamin levels in the body, or that an inadequate intake would necessarily result in inadequate blood serum levels. Vitamin blood serum concentrations are also dependent on a series of other complex factors such as an individual’s capacity of absorption or the bioavailability of the specific vitamin depending on the form that is ingested, among others. To indicate the effect of cancer treatment on vitamin levels in the body, it would be necessary to collect blood samples at the same time the dietary survey is administered, and the results could be an important source of information when designing an individualized nutritional intervention plan during and after treatment.

## 5. Conclusions

The inadequacy of vitamin intake during and after treatment suggests an inadequate diet but these results need to be confirmed by further studies such as larger prospective cohorts and randomized controlled trials. Taking into account that in the women participating in this study no nutritional intervention has been done, it would be convenient to evaluate this aspect to improve the recovery after treatment, diminish the toxicity of the treatment and improve the lifestyle.

An early nutritional intervention and nutritional assessment at all stages of treatment could increase life expectancy and decrease the incidence of breast cancer mortality. For this, it is necessary to evaluate the diet as a whole: macro and micronutrients to be able to establish the adequate nutritional therapy in the case of these patients. Also, and given the result that show that during treatment there is a change in the dietary pattern of the patients, exploring more in depth the reasons why this change appears is the logical next step in order to help establish this nutritional intervention and support plan. 

## Figures and Tables

**Table 1:** Main characteristics of the breast cancer’s cohort.

	Mean ± SD	Minimum	Maximum
Age	51.49 ± 11.17	30	79
Weight (kg)	65.32 ± 9.80	50	86
Height (cm)	160.56 ± 6.51	146	178
BMI (kg/m^2^)	25.52 ± 4.83	18.9	35.8
PAL	1.48 ± 0.19	1.2	1.64

BMI: body mass index; PAL: physical activity level.

**Table 2:** Vitamin intake in the three stages of the study.

Vitamin	UL	DRI	EAR	Intake	Mean ± SD (Min–Max)	*p*-Value	% EAR (Min–Max)	*p*-Value
A	3000 µg/day	700 µg/day	500 µg/day	Before	998.68 ± 440.88 (427.00–2116)	0.140	199.73 ± 88.18 (85.40–423.20)	0.140
				During	1148.24 ± 616.97 (315.00–3113.00)		229.65 ± 123.39 (63.00–622.60)	
				After	932.35 ± 326.21 (395–1725)		186.47 ± 65.24 (79.00–345.00)	
D	100 µg/day	15 µg/day	10 µg/day	Before	3.53 ± 2.55 (0.25–12.20)	0.182	35.28 ± 25.55 (2.50–132.00)	0.182
				During	3.60 ± 2.84 (0.20–11.10)		35.96 ± 28.43 (2.00–111.00)	
				After	2.63 ± 2.0 (0.11–8.60)		26.30 ± 20.69 (1.10–86.00)	
E	1000 mg/day	15 mg/day	12 mg/day	Before	9.56 ± 3.46 (4.20-20.30)	0.211	79.70 ± 28.81 (35.00–169.17)	0.211
				During	9.05 ± 3.97 (3.60–21.70)		75.45 ± 33.08 (30.00–180.83)	
				After	8.13 ± 3.13 (3.40–16.10)		67.77 ± 26.08 (28.33–134.17)	
K	ND	90 µg/day (AI)	90 µg/day (AI)	Before	156.51 ± 96.60 (31.40–522.00)	0.520	173.90 ± 107.34 (34.89–580.00)	0.520
				During	142.01 ± 67.36 (49.40–283.00)		157.80 ± 74.84 (54.89–314.44)	
				After	134.94 ± 81.27 (34.60–393.00)		149.93 ± 90.30 (38.44–436.67)	
B_1_	ND	1.1 mg/day	0.9 mg/day	Before	1.44 ± 0.60 (0.60–3.70)	0.113	159.58 ± 67.72 (66.67–411.11)	0.113
				During	1.40 ± 0.45 (0.69–2.60)		155.07 ± 50.55 (76.67–288.89)	
				After	1.22 ± 0.34 (0.74–2.00)		135.88 ± 37.37 (82.22–222.22)	
B_2_	ND	1.1 mg/day	0.9 mg/day	Before	1.90 ± 0.74 (0.81–4.40)	0.006	211.23 ± 82.32 (90.00–488.89)	0.006
				During	1.68 ± 0.55 (0.73–3.20)		186.63 ± 61.44 (81.11–355.56)	
				After	1.46 ± 0.40 (0.71–2.80)		161.89 ± 44.02 (78.89–311.11)	
B_3_	35 mg/day	14 mg/day	11 mg/day	Before	37.19 ± 9.17 (21.80–70.40)	0.042	338.10 ± 83.38 (198.18–640.00)	0.042
				During	35.27 ± 7.78 (15.80–53.90)		320.63 ± 70.69 (143.64–490.00)	
				After	32.45 ± 7.61 (18.90–48.90)		295.01 ± 69.22 (171.82–444.55)	
B_5_	ND	5 mg/day (AI)	5 mg/day (AI)	Before	5.37 ± 1.04 (2.80–7.30)	0.001	107.41 ± 20.70 (56.00–146.00)	0.001
				During	4.85 ± 1.06 (2.60–6.90)		97.02 ± 21.10 (52.00–138.00)	
				After	4.53 ± 0.88 (3.10–6.90)		90.65 ± 17.75 (62.00–138.00)	
B_6_	100 mg/day	1.4 mg/day	1.4 mg/day	Before	2.30 ± 0.84 (1.20–5.60)	0.450	164.09 ± 60.36 (85.71–400)	0.450
				During	2.21 ± 0.58 (1.10–3.80)		158.10 ± 41.96 (78.57–271.43)	
				After	2.10 ± 0.59 (0.98–3.50)		149.96 ± 42.26 (70.00–250.00)	
B_8_	ND	30 µg/day (AI)	30 µg/day (AI)	Before	29.49 ± 8.93 (10.10–53.80)	0.021	98.31 ± 29.78 (33.67–179.33)	0.021
				During	26.39 ± 8.90 (8.00–47.70)		87.99 ± 29.66 (26.67–159.00)	
				After	23.99 ± 7.04 (12.50–40.90)		79.97 ± 23.45 (41.67–136.33)	
B_9_	1000 µg/day	400 µg/day	320 µg/day	Before	280.03 ± 103.70 (120.00–586.00)	0.462	87.50 ± 32.40 (37.50–183.13)	0.462
				During	273.08 ± 95.57 (133.00–526.00)		85.34 ± 29.87 (41.56–164.38)	
				After	255.03 ± 65.90 (129.00–455.00)		79.69 ± 20.59 (40.31–142.19)	
B_12_	ND	2.4 µg/day	2 µg/day	Before	5.88 ± 2.74 (1.30–14.40)	0.143	294.05 ± 136.80 (65.00–720.00)	0.143
				During	6.57 ± 6.70 (0.72–34.50)		328.71 ± 335.50 (36.00–1725.00)	
				After	4.58 ± 2.38 (1.80–11.10)		229.05 ± 119.28 (90.00–555.00)	
C	2000 mg/day	75 mg/day	60 mg/day	Before	137.04 ± 65.04 (30.70–319.00)	0.940	228.41 ± 108.41 (51.17–531.67)	0.940
				During	141.99 ± 65.40 (44.60–379.00)		236.64 ± 109.00 (74.33–631.67)	
				After	139.52 ± 51.17 (13.40–238.00)		232.53 ± 85.28 (22.33–396.67)	

UL: Upper Level; DRI: Dietary Reference Intake; EAR: Estimated Average Requirements.

**Table 3:** Relative risk that the consumption of vitamins is lower than the EAR in the three stages of the study.

Vitamin	EAR	Intake	<EAR (%)	*p*-Value	HRc (CI 95%)	Trends *p*-Value
A	500 µg/day	Before	5.4	0.173	1 (Ref.)	0.512
		During	13.5		1.50 (0.44–5.10)	
		After	2.7		1.50 (0.45–5.05)	
D	10 µg/day	Before	97.3	-	1 (Ref.)	0.989
		During	97.3		1.00 (0.95–1.06)	
		After	100		1.00 (0.95–1.06)	
E	12 mg/day	Before	78.4	0.783	1 (Ref.)	0.404
		During	83.8		1.07 (0.91–1.25)	
		After	83.8		1.07 (0.91–1.25)	
K	90 µg/day (AI)	Before	24.3	0.452	1 (Ref.)	0.152
		During	32.4		1.44 (0.97–2.40)	
		After	37.8		1.44 (0.87–2.39)	
B_1_	0.9 mg/day	Before	16.2	0.451	1 (Ref.)	0.843
		During	10.8		1.11 (0.46–2.66)	
		After	21.6		1.09 (0.45–2.66)	
B_2_	0.9 mg/day	Before	2.7	0.771	1 (Ref.)	0.651
		During	5.4		1.52 (0.25–9.37)	
		After	2.7		1.52 (0.25–9.37)	
B_3_	11 mg/day	Before	0	-	1 (Ref.)	-
		During	0		-	
		After	0		-	
B_5_	5 mg/day (AI)	Before	31.4	0.002	1 (Ref.)	0.001
		During	54.1		4.11 (2.07–8.18)	
		After	73.0		4.11 (2.10–8.08)	
B_6_	1.4 mg/day	Before	2.9	0.811	1 (Ref.)	0.407
		During	5.6		2.06 (0.4–11.5)	
		After	5.4		2.06 (0.4–11.5)	
B_8_	30 µg/day (AI)	Before	5.4	0.001	1 (Ref.)	0.001
		During	100		18.96 (6.28–57.2)	
		After	5.4		18.73 (8.45–41.4)	
B_9_	320 µg/day	Before	73.0	0.148	1 (Ref.)	0.359
		During	67.6		1.24 (0.59–2.62)	
		After	86.5		1.24 (0.59–2.62)	
B_12_	2 µg/day	Before	2.7	0.001	1 (Ref.)	0.001
		During	100		43.41 (9.91–140)	
		After	10.8		42.91 (16.89–108)	
C	60 mg/day	Before	10.8	0.695	1 (Ref.)	0.386
		During	8.1		0.60 (0.19–1.92)	
		After	5.4		0.60 (0.19–1.92)	

EAR: Estimated Average Requirements; OR: Odds ratio; CI: Confidence Interval; HRc: Hazard ratio.

## Data Availability

The data presented in this study are available on request from the corresponding author. The data are not publicly available due to patient privacy.
